# Culture-detected urinary bacteria 10–20 years after tension-free vaginal tape surgery and associations with incontinence, pelvic pain, and dissatisfaction: a cross-sectional study

**DOI:** 10.1186/s12894-026-02181-7

**Published:** 2026-05-18

**Authors:** Ingrid Volløyhaug, Berit R Solhaug, Kjersti W Larssen, Kjersti Haugum, Maria Ø Nyhus, Rune Svenningsen, Jan E Afset

**Affiliations:** 1 Department of Obstetrics and Gynecology, St. Olavs Hospital HF, Trondheim University Hospital , Postboks 3250, Torgarden, Trondheim, N-7006 Norway; 2 Department of Clinical and Molecular Medicine, Norwegian University of Science and Technology, Trondheim, Norway; 3 Department of Medical Microbiology, St. Olavs Hospital, Trondheim University Hospital, Trondheim, Norway; 4 Faculty of Medicine, University of Oslo, Oslo, Norway; 5 Department of Gynecology, Oslo University Hospital, Oslo, Norway; 6 The Norwegian Female Incontinence Registry, Oslo University Hospital, Oslo, Norway

**Keywords:** Pelvic pain, Stress urinary incontinence, Tension free vaginal tape, Urgency urinary incontinence, Urinary microbiota

## Abstract

**Background:**

Some women have persistent incontinence or pain after tension-free vaginal tape (TVT) surgery. Our objective was to study any association between culture-detected bacteriuria and incontinence, complications and satisfaction 10–20 years after TVT.

**Methods:**

Cross-sectional study in 2022 of 127 women operated with TVT between 2001-2012 at Trondheim University Hospital, Norway. Validated questionnaires assessed stress urinary incontinence (SUI), urgency urinary incontinence (UUI), pelvic pain and satisfaction. Urine collected by sterile catheterization was cultivated for two (chromogenic and blood agar) and five days (blood, fastidious and chocolate agar). Symptoms and demographics were compared between women with and without significant bacterial growth, defined as ≥ 1.000 CFU/ml for this study, using chi-square test, Fishers exact test, t-test and logistic regression. Number of species detected after five days culture was tested in association to demographics and symptoms using Mann Whitney-U-test.

**Results:**

50/111 women (45.0%) had SUI, 54/122 (44.3%) had UUI, 95/116 (81.9%) were satisfied and 7/123 (5.7%) stated persistent pain. 16 (12.4%) had significant bacterial growth after two days of whom seven (44%) had a positive dipstick screening test (leukocytes or nitrite). After five days culture, 28 (22%) had significant bacterial growth. We found non-significantly higher odds for SUI (aOR 4.2, 95% CI 0.5–36.1) and UUI (aOR 2.1, 95% CI 0.3–13.7) in women with bacterial growth after two days. Bacterial growth after five days was associated with non-significantly increased risk of SUI (aOR 2.3, 95% CI 0.6–8.9) and UUI (aOR 3.4, 95% CI 0.9–13.6). Age was significantly associated with bacterial growth after two (aOR 1.2, 95% CI 1.02–1.3) and five days (aOR 1.1, 95% CI 1.04–1.2). The number of bacterial species after five days culture was higher in women with UUI, BMI > 75 percentile, in those dissatisfied or with persistent pain, than in women without these conditions (*p* < 0.01).

**Conclusions:**

Significant bacteriuria and larger variety of bacterial species were associated with incontinence, pain and dissatisfaction 10–20 years after TVT. The dipstick screening test had a sensitivity of only 44%. Extended urinary culture may be of value in optimizing treatment of urinary tract symptoms after TVT.

**Trial registration:**

ClinicalTrials.gov (NCT04912830).

**Supplementary Information:**

The online version contains supplementary material available at 10.1186/s12894-026-02181-7.

## Background

Tension free vaginal tape (TVT) surgery is associated with high subjective and objective cure for women with stress urinary incontinence (SUI) [1–3]. However, there has been concern regarding long-term complications, such as persisting pelvic pain, erosions of the polypropylene mesh and dissatisfaction after TVT. Over-weight and mixed urinary incontinence prior to surgery are associated with persisting symptoms and lower satisfaction after TVT [1]. Local estrogens can be used to prevent urinary tract infections (UTI) that contribute to urgency, pain and incontinence after surgery [4]. TVT removal has been an alternative for patients with persistent pain, but this procedure is associated with complications and recurrent incontinence [5]. Still, much is not known about modifiable factors that could be targeted to improve long-term outcome after TVT.

Until 2012, the urinary tract of healthy individuals was considered to be a sterile environment [6]. Over the past decade, after the discovery of metagenomic sequencing revealed a unique urinary microbiome, much research has focused on urinary microbiota in relation to lower urinary tract symptoms. A review from 2022 suggested a potential relationship among gut, vaginal, and urinary microbiomes in women with over active bladder [7]. Bacterial diversity is different between continent and incontinent women and in individuals with and without pain in the lower urinary tract [8]. Still, there is a limited number of studies on this topic, and the role of microbiota and culture-detected bacteriuria in long-term symptoms and satisfaction after TVT using polypropylene mesh has not been properly explored. If culture-detected urinary bacteria in women referred with problems after TVT is associated with symptoms such as incontinence, pain and dissatisfaction, this is a possible target for intervention.

Metagenomic sequencing is still too time-consuming and expensive for use in clinical practice. Dipstick testing is often used to screen women with symptoms after TVT, and specimens with a positive dipstick test are examined with a standard two-day urine culture. The accuracy of dipstick testing in detecting UTI depends on the presence of symptoms and population, e.g. pregnant or elderly women [9]. If we find that standard and extended urinary culture of dipstick negative urine in women with pain, incontinence and dissatisfaction after TVT is different than in cured and satisfied women, this may help improve investigation guidelines, treatment options and improve long-term results after TVT.

Our objective was therefore to study the presence of potential urinary tract pathogens after two days standard culture and five days extended culture of sterile catheter urine in association with incontinence, long-term pain and satisfaction in women more than 10 years after their TVT surgery. We also aimed to test if a urinary dipstick test was sufficient to screen for significant bacteriuria in women after TVT.

## Material and methods

This was a cross-sectional study conducted in 2022 at Trondheim University Hospital, Norway, with the aim to study the presence and association of potential urinary tract pathogens after two- and five-days culture of urine collected by sterile catheterization with incontinence, long-term pain and satisfaction after TVT surgery.

### Participants

A total of 153 women underwent TVT surgery between 2001-2006 and 2011–2012 at Trondheim University Hospital and they were all invited to participate. This was part of a larger study including women operated at 19 hospitals in Norway, where the primary aim was to determine the rates of subjective and objective cure, satisfaction, and pain 10 and 20 years after TVT. Results of the parent study have already been published [1]. In the present study, all women operated at Trondheim University Hospital between 2001 and 2006 who were still alive and had an address in Norway in 2022 were eligible. For the time frame 2011–2012, only women younger than 42 years at time of surgery were invited, to ensure a substantial part of premenopausal women when the study was conducted in 2022. Older women from that period were not invited to avoid overshooting the power estimate of the parent study. Exclusion criteria were not being able to communicate in Norwegian or English, deceased or emigrated out of Norway. The Gynecare TVT^®^ (Johnson & Johnson) was the only option for the 2001–2006 cohort, and in 2011–2012 also the Gynecare TVT Obturator System^®^ (Johnson & Johnson) was available. No mini-slings were used. The study was approved by the Regional Committee for Medical and Health Research Ethics in Mid-Norway (REK Midt 2020/141799) and registered with ClinicalTrials.gov (NCT04912830). All women had signed an informed consent.

### Data sources

The women filled out a validated questionnaire for assessment of SUI, UUI and satisfaction with TVT [10]. This generated a SUI index score with a range of 0–12 and an UUI index score with a range of 0–8 where higher scores indicate more bothersome symptoms. A SUI index < 3 was defined as cured according to the definition used in the Norwegian Female Incontinence Registry [11]. We calculated the proportion of women with SUI index ≥ 3 (persistent or recurrent SUI) and the proportion with SUI index > 3, as the former definition seemed to be too strict according to the findings in the parent study [1]. We applied an UUI index ≥ 3 as cut-off for bothersome persistent or de novo UUI. Women graded their treatment satisfaction using a five-point Likert scale, ranging from “very satisfied”’ to “very unsatisfied”. The categories “very satisfied” and “satisfied” were merged into “satisfied”, and “very unsatisfied”, “unsatisfied” and “no change” were counted as “dissatisfied”. Women were also asked if they had persistent pelvic pain that they related to the TVT surgery, where the answer options were “yes” or “no”.

Recurrent UTI (> 1 over last year) was reported by the women filling out the questionnaire. Any use of antibiotics, Methenamine hippurat (Hiprex^®^) or probiotics as UTI prophylaxis in 2022 was registered. Use of hormones was difficult to interpret, as women did not distinguish clearly between contraceptives and menopausal hormonal replacement therapy when responding to this question and this variable was therefore not used in the analyses.

Study participants were invited for a clinical exam and asked to meet with full bladder and urinate immediately before the exam. Post void residual urine was measured using sterile catheterization to empty the bladder, and a residual ≥ 200 ml was counted as significant. The variable “residual urine” was registered only for women who had at least 200 ml in the bladder before voiding. The urine sample was tested for leucocytes and nitrite by a dipstick test (Combur-6-test, Roche), and samples positive for nitrite or leucocytes (≥ 10 × 10^6^/L) were registered as “dipstick positive”. All urine samples, regardless of reported symptoms from urinary tract/lower abdomen, dipstick result or whether the woman was using antibiotics or other UTI prophylaxis, were cultivated for two days on chromogenic agar and blood agar. In addition, extended culture was performed for five days on blood agar, chocolate agar and fastidious anaerobic agar as detailed in Supplement 1.

All bacterial growth registered at day 1, 2 and 5 was analyzed by Maldi-TOF MS using MALDI Biotyper Compass software (version 11.0.0.0_9607–10833, Bruker Daltonics, Germany). A score value ≥ 2.0 was regarded as valid for identification to species level and a score value 1.7 - < 2.0 was regarded as valid for identification to genus level according to manufacturer´s recommendations. A score value 1.7 - < 2.0 was also accepted for identification to species level if the first four suggestions by the Compass software were consistent and the distance to the next suggested species was > 0.3 according to standard operating procedure of the laboratory [12, 13].

Growth of each species was quantified as < 10^2^, ≥10^2^-<10^3^, ≥10^3^- <10^4^, ≥ 10^4^- <10^5^, ≥ 10^5^ colony forming units (CFU)/mL urine. For the purpose of this study, bacterial growth after two days standard culture of ≥ 10^3^ CFU/mL each of 1–2 species of urinary tract pathogens (e.g. *Escherichia coli*,* Enterococcus faecalis* or *Aerococcus urinae*) was classified as significant bacterial growth, in line with European guidelines for microbiological diagnosis of symptomatic urinary tract infection when urine is obtained by catheterization [14]. The low threshold for significant bacterial growth was chosen to allow detection of low-level bacterial growth that may be relevant in studies of urinary microbiota and long-term lower urinary tract symptoms, even when classical diagnostic criteria for symptomatic UTI are not met (Supplement 2). Growth of three species or more was regarded as mixed culture. Classification of doubtful pathogens and commensal genital flora was done according to the same guidelines [14]. After five days extended urinary culture, growth of ≥ 10^3^ CFU/mL each of primary, secondary and doubtful pathogens in pure or mixed culture, but not commensal genital flora, was classified as significant growth. In addition, we assessed the total number of species identified after five days culture within different bacterial phyla from each urinary sample, regardless of quantity in CFU/mL.

### Statistics

Statistics were performed using SPSS 30.0.0.0 (172) (IBM. Armonk, NY, USA). Persistent or de novo SUI, UUI, persistent pain and satisfaction were primary outcomes.

UTI prophylaxis, residual urine, age in 2022 and self-reported BMI were treated as possible confounders. Time since surgery was strongly correlated to age in 2022 and therefore not entered as an independent variable. Continuous variables were tested for normality. If data were missing for some of the variables, we only used data for respondents, and no imputation was performed. Dichotomous variables were compared between women with and without significant bacterial growth of common urinary tract pathogens after two and five days using the X^2^ test or Fisher’s exact test when appropriate, and continuous variables using independent samples t-test. Then we performed a multiple logistic regression analysis for the outcomes SUI ≥ 3 and UUI ≥ 3, correcting for the possible confounders age, BMI, UTI prophylaxis and residual urine ≥ 200 ml. Only a small proportion of women reported persistent pain or dissatisfaction, and multivariable analysis was therefore not possible for these outcomes.

The number of species within different bacterial *phyla* after five days of culture was tested in relation to symptoms, satisfaction, residual urine ≥ 200 ml, age (> 52 years as estimate for menopause) and BMI (> 25 kg/m^2^ and > 75 percentile) using the Mann Whitney-U-test. Any correlation between number of species, age and BMI was tested with Spearman’s rank correlation. A *p*-value < 0.05 was counted as statistically significant. Power calculations were performed for the parent study and not for the variables tested in this study.

## Results

A total of 127 of 153 (83.1%) invited women were examined on average (SD) 16.3 (4.5) years after TVT surgery. Fifteen (13.5%) had a TVT obturator sling, the remaining 96 (86.5%) were retropubic slings. Mean (SD) age was 60.0 (10.7) years, BMI was 27.6 (4.9) kg/m^2^ and 75/120 (62.5%) of the women were over-weight with a BMI > 25 kg/m^2^. A total of 38/120 (31.7%) had BMI > 75th percentile (BMI > 29 kg/m^2^). Fifty of 111 women (45.0%) had SUI index ≥ 3, 357,111 (31.5%) SUI index > 3, 54/122 (44.3%) had UUI index ≥ 3, 95/116 (81.9%) were satisfied and 7/123 (5.7%) stated persistent pain after TVT. Eighteen of 125 (14.4%) had recurrent UTI (> 1 over last year), and 16/127 (12.6%) used UTI prophylaxis (antibiotics, Methenamine hippurate (Hiprex^®^) or probiotics).

We found significant bacterial growth (≥ 10^3^ CFU/mL) of *E. coli*,* E. faecalis* or *A. urinae* in the urine sample from 16 (12.6%) of the women after two days. Seven of these had a positive dipstick test (leukocytes or nitrite), giving a sensitivity of 44% (7/16) and positive predictive value of 58% (7/12). Five had a positive dipstick test that turned out negative after culture, giving a specificity of 95% (106/111) and negative predictive value of 92% (106/115) of the dipstick screening test. On univariate analysis, women with significant bacterial growth after two days standard culture had higher prevalence of SUI index ≥ 3, UUI index ≥ 3 and dissatisfaction with TVT compared to women with no or non-significant bacterial growth, but the differences did not reach statistical significance (Table 1). Women with bacteriuria were significantly older and residual urine ≥ 200 ml was significantly more common (Table 1).

**Table 1:** Prevalence of symptoms and complications in women with and without 1) significant growth (10^3^ CFU/mL) of primary and secondary urinary pathogens after two days standard culture, and 2) significant growth (10^3^ CFU/mL) of primary, secondary and doubtful pathogens (excluding commensal genital flora) in pure or mixed culture after five days extended culture. Comparison of women with and without significant bacterial growth

	Two days standard urine culture				Five days extended urine culture			
	Significant bacterial growth (*n* = 16)	No or non-significant bacterial growth (*n* = 111)			Significant bacterial growth (*n* = 28)	No or non-significant bacterial growth (*n* = 99)		
Primary outcomes	*n/N (%)*	*n/N (%)*	*Odds ratio* *95% CI*	*P*	*n/N (%)*	*n/N (%)*	*Odds ratio* *95% CI*	*P*
SUI index ≥3^a^ (*n* = 50/111)	7/12 (58.3%)	43/99 (43.4%)	1.8 (0.5–6.1)	0.33^d^	15/23 (65.2%)	35/88 (39.8%)	2.8 (1.1–7.4)	0.03 ^d^
UUI index ≥3^b^ (*n* = 54/122)	7/15 (53.3%)	46/107 (43.0%)	1.5 (0.5–4.5)	0.58^d^	14/26 (53.8%)	40/96 (41.7%)	1.8 (0.7–3.9)	0.28 ^d^
Dissatisfied with TVT (*n* = 21/116)	4/13 (30.7%)	17/103 (16.5%)	2.2 (0.6–8.1)	0.25^e^	8/23 (34.8%)	13/93 (14.0%)	3.2 (1.2–9.3)	0.03^e^
Persistent pain after TVT (*n* = 7/123)	1/15 (6.7%)	6/108 (5.6%)	1.2 (0.1–10.8)	1.0^e^	1/26 (3.8%)	6/97 (6.2%)	0.6 (0.1–5.2)	1.0^e^
Possible explanatory variables								
Residual urine ≥ 200 ml (*n* = 8/90)	4/10 (40.0%)	4/80 (5.0%)	12.7 (2.5–63.8)	< 0.01^e^	4/18 (22.2%)	4/72 (5.6%)	4.9 (1.1–21.8)	0.048^e^
UTI prophylaxis^c^ (*n* = 16/127)	4/16 (25.0%)	12/111 (10.8%)	2.8 (0.8–9.9)	0.12^e^	7/28 (25.0%)	9/99 (9.1%)	3.3 (1.1–10.0)	0.047^e^
	*Mean (SD) *	*Mean (SD)*	*Mean Difference* *95% CI*	*P* ^*f*^	*Mean (SD) *	*Mean (SD)*	*Mean Difference* *95% CI*	*P* ^*f*^
Age, years (*n* = 127)	71.5 (10.5)	58.3 (9.7)	13.2 (8.0-18.4)	< 0.01	69.6 (10.1)	57.2 (9.2)	12.4 (8.6–16.4)	< 0.01
BMI, kg/m^2^ (*n* = 120)	27.2 (5.5)	27.6 (4.9)	-0.4 (-3.3-2.5)	0.80	27.8 (5.3)	27.5 (4.9)	0.4 (-1.9-2.6)	0.75

a Stress urinary incontinence (SUI) index range 0–12, SUI index ≥ 3 is not subjectively cured

b Urgency urinary incontinence (UUI) index range 0–8, UUI index ≥ 3 is significant UUI

c Urinary tract infection (UTI) prophylaxis is any prophylactic use of antibiotics, Methenamine hippurate (Hiprex^®^) or probiotics in women with recurrent UTI

d Chi-square test

e Fisher’s Exact test

f Independent samples t-test

After five days of culture, 28 (22%) of the women had significant growth of primary, secondary or doubtful pathogens, 80 (63%) had growth of commensal genital flora and 19 (15%) had no growth. Women with significant bacterial growth of primary, secondary or doubtful pathogens (excluding commensal genital flora) after five days had a three-fold increased risk of SUI index ≥ 3 and dissatisfaction with TVT, and residual urine ≥ 200 ml was more common (Table 1). UTI prophylaxis was associated with increased risk of significant growth of bacterial pathogens after five days culture (Table 1). Women with significant bacterial growth were on average 12–13 years older than women without or non-significant bacterial growth (Table 1).

On multivariate analysis, there was a tendency to higher odds for SUI ≥ 3 (aOR 4.2, 95% CI 0.5–36.1, *p* = 0.19) and UUI ≥ 3 (aOR 2.1, 95% CI 0.3–13.7, *p* = 0.4), but only age was significantly associated with growth of primary or secondary pathogens after two days culture (aOR 1.2, 95% CI 1.0–1.3, *p* = 0.02). In women with significant growth of primary, secondary or doubtful pathogens after five days culture, we also observed a non-significant increased risk of SUI ≥ 3 (aOR 2.3, 95% CI 0.6–8.9, *p* = 0.22) and UUI ≥ 3 (aOR 3.4, 95% CI 0.9–13.6, *p* = 0.08), but age was the only variable significantly associated with bacterial growth (aOR 1.1, 95% CI 1.0–1.2, *p* < 0.01).

One-hundred-eight women (85%) had growth of any kind of bacteria including commensal genital flora after five days, with a mean number of species for each woman of 2 (range 0–6). Different species belonging to the phyla *Actinomycetota* (*n* = 25), *Bacillota* (*n* = 31), *Bacteriodota* (*n* = 5), *Fusobacteria* (*n* = 1), *Proteobacteria* (*n* = 6) and one *Ascomycota* (*n* = 1) were found. Overweight women and women with SUI index ≥ 3 had growth of a higher number of bacterial species than normal weight women and women with SUI index < 3, but this did not reach statistical significance (Table 2). Women with either UUI index ≥ 3, BMI > 75 percentile, those who were dissatisfied with TVT or had persistent pain after TVT had a significantly larger diversity of bacterial species in their urine sample than women without these conditions (Table 2). We also found a weak positive correlation between BMI and number of species (r_s_ = 0.21, *p* = 0.02), whereas age as continuous variable was not significantly correlated with the number of species (r_s_ = 0.11, *p* = 0.23). 

**Table 2:** Mean (SD) and median (range) number of bacterial phyla after five days extended culture for women with and without symptoms and conditions. All bacterial species identified including primary, secondary, doubtful pathogens and commensal genital flora irrespective of colony count were included in this analysis

Symptom or condition	Yes(symptom/ condition present)	No(symptom/ condition not present)	
	Mean (SD), Median (range) Number of bacterial phyla		*P*-value^a^
Stress urinary incontinence index ≥3^b^	2.3 (1.6), 2 (0-6)	1.8 (1.5), 1 (0-6)	0.07
Urgency urinary incontinence index ≥3^c^	2.3 (1.5), 2 (0-6)	1.7 (1.5), 1 (0-6)	0.01
Dissatisfied with TVT^d^	2.8 (1.4), 2 (1-6)	1.7 (1.4), 1 (0-6)	<0.01
Persistent pain after TVT^e^	2.9 (1.1), 3 (2-5)	1.9 (1.6), 2 (0-6)	0.03
Residual urine ≥ 200 ml^f^	1.6 (1.1), 2 (0-6)	1.9 (1.6), 1.5 (0-3)	0.91
Age > 52 years (estimated menopause)^g^	2.1 (1.7), 2 (0-6)	1.7 (1.3), 1 (0-5)	0.20
Overweight (BMI > 25 kg/m^2^)^h^	2.1 (1.6), 2 (0-6)	1.8 (1.5), 1 (0-6)	0.30
BMI over 75 percentile (BMI > 29 kg/m^2^)^i^	2.5 (1.8), 2 (0-6)	1.7 (1.4), 1.5 (0-6)	0.04
Urinary tract infection prophylaxis^j^	2.1 (1.7), 1.5 (0-5)	1.9 (1.5), 2 (0-6)	0.89

a Mann-Whitney U-test

b Stress urinary incontinence index ≥ 3 50/111 (45.0%),

c Urgency urinary incontinence index ≥ 3 54/122 (44.3%),

d Dissatisfied with TVT 21/116 (18,1%),

e Persistent pain after TVT 7/123 (5.7%),

f Residual urine ≥ 200 ml 8/90 (8.9%),

g Age > 52 years 84/127 (66.1%),

h BMI > 25 kg/m^2^ 75/120 (62.5%),

i BMI > 75^th^ percentile (BMI > 29 kg/m^2^) 38/120 (31.7%),

j Urinary tract infection prophylaxis (antibiotics, Methenamine hippurate (Hiprex®) or probiotics) 16/127 (12.6%)

## Discussion

### Principal findings

In women followed 10–20 years after TVT surgery, we found that significant growth of urinary pathogens after two and five days urine culture was associated with higher age. We also observed an increased odds of SUI ≥ 3 and UUI ≥ 3 after two and five days, but these findings were not statistically significant. UUI, persistent pelvic pain and dissatisfaction were associated with growth of a greater variety of potential pathogenic bacteria after five days urine culture.

### Results in context of what is known

The results from our cohort of women examined on average 16 years after TVT corroborate with other studies of women from general populations using metagenomic testing. It is well known from several epidemiologic studies that the prevalence of urinary incontinence and especially UUI increases with advancing age [15, 16]. Some studies have found that bacterial diversity increases after menopause [17, 18]. We know that older women have lower estrogen levels, which may be part of the explanation. In the present study, we found no linear correlation between age and number of bacterial species, but women with bacteriuria after two- and five-days culture were 12–13 years older than those with no growth, supporting that menopause represents a threshold and is the strongest contributor to the age effect. A substantial proportion of women in the general population has asymptomatic bacteriuria, also increasing with advancing age, which is similar to our results [17, 18]. Previous studies have found that systemic hormones may cause or worsen existing urinary symptoms whereas vaginal estrogen improves dysuria, UUI, SUI and prevents recurrent UTI in postmenopausal women [19]. Unfortunately, we had no reliable information on hormonal use in this study. In a previous publication from our study population, we found that older women were less satisfied after TVT [1]. Higher prevalence of bacteriuria in older women may therefore have contributed to this.

We found a weak positive correlation between increasing BMI and bacterial diversity. When using BMI 25 kg/m^2^ as cut off, we found a non-significantly higher number of bacterial species in overweight women. When applying a higher cut-off, we found that obese women with BMI over the 75th percentile (BMI > 29 kg/m^2^ in this population) had a significantly higher number of bacteria species. This also corroborates with other studies using metagenomic testing, where they found that bacterial diversity was significantly associated with higher BMI [20, 21]. Greater bacterial diversity might be part of the explanation why women with high BMI are less satisfied after TVT [1], adding to the obvious mechanical effect of high BMI on urinary incontinence.

In the present study, a post void residual urine ≥ 200 ml was linked with bacteriuria. It is well established that incomplete voiding represents the primary risk factor for UTIs associated with both urinary incontinence and prolapse [22]. Clean intermittent catheterization is a treatment option for women with recurrent UTI due to high residual urine, but in women who have undergone TVT, cutting of a tight tape may be necessary to avoid UTI and other symptoms. We found that women on UTI prophylaxis had higher risk of significant bacteriuria. A likely explanation to this paradox is that only women with an established problem of recurrent UTI would be using UTI prophylaxis at long-term follow up after TVT. Without prophylaxis, an even higher proportion of them could have had significant bacteriuria.

In the multivariate analysis, we observed higher odds of SUI index ≥ 3 in women with significant bacterial growth after two- and five-days culture, but this did not reach statistical significance. This link between SUI and bacterial growth was not found in most previous studies [20, 23, 24]. Thomas-White et al. examined women prior to SUI surgery using a different questionnaire than in the present study and these factors may explain why their results differ from our results from a study population examined many years after TVT [16]. Similar to our findings, a more recent study using metagenomic testing found that the composition of the bladder microbiome was associated with urinary incontinence, regardless of incontinence subtype [8]. In the multivariate analysis, we also observed higher odds of UUI index ≥ 3 in women with significant bacterial growth after two- and five-days culture, but this association did not reach statistical significance. The link between UUI and urinary microbiome was found in several previous studies [21, 24]. A review from 2019 found that higher bacterial diversity in the absence of *Lactobacillus* dominance was associated with UUI and resistance to anticholinergic treatment [25]. Chronic low grade bacterial colonization of the urinary bladder may exacerbate UUI and explain why anticholinergic and muscarinic treatment are not always successful [26]. The results of our study in combination with previous studies suggest that microorganisms in the urine may be an important target in treatment of UUI in women after TVT.

In the present study, women with persistent pain more than 10 years after TVT had a higher number of bacterial species in the urine than women without pain. Another recent study found similarly that gut, vaginal, and urinary microbiota are biomarkers of sensitization in women with chronic pelvic pain [27]. Fear of pain after TVT has led to discontinuation of its use in some countries, and many women have had their TVT’s removed because of pain. Some studies have shown durable pain relief in women who have had TVT removal [28], but we also know that partial, and complete tape removal often lead to recurrence of SUI [29]. If specific bacterial signatures in the urine, gut and vagina can explain some of the pain sensitization in TVT patients, we believe this could lead to different strategy for women with pain after TVT.

### Clinical implications

Important clinical implications of our findings are that only 44% of the women with significant bacterial growth after two days urine culture had a positive urine dipstick screening test. This indicates that women with incontinence symptoms, pain or dissatisfaction after TVT should have a urine culture regardless of the result of the dipstick screening test. Extended urine culture for five days can be useful in women with pain and dissatisfaction after TVT, as this may help to identify potentially pathogenic bacteria not detected at dipstick screening or culture of shorter duration. Growth of a wider diversity of bacteria in women with symptoms may be part of the explanation to persistent or de novo incontinence, pain and dissatisfaction.

### Research implications

It remains to investigate if antibiotics targeting urinary pathogens have an effect in treating women who are dissatisfied, have UUI or pain after TVT. Investigation of culture-detectable urinary bacteria in women who are dissatisfied after TVT, and subsequent targeted treatment could be feasible prior to more radical interventions such as tape removal, medical treatment of UUI, botulinum toxin injections or repeat incontinence surgery. Since persistent pelvic pain was infrequent in this study, larger scale studies properly powered to investigate associations between dissatisfaction, incontinence, pain and culture-detected urinary bacteria are needed to confirm our results.

### Strengths and limitations

The main strength of this study is the long follow up of women after TVT. To our knowledge, no previous studies have assessed the association between urinary microbiota, patient satisfaction and incontinence symptoms this many years after TVT. Furthermore, we used a validated questionnaire to assess SUI, UUI and treatment satisfaction. Post void urine collected by sterile catheterization was used for dipstick testing and urine culture, eliminating contamination from the urethra, vagina and vestibulum. The urine culture was assessed for bacterial growth after two days and five days, making it possible to evaluate additional value of extended culture. It is well known that slow-growing potential uropathogens fail to grow after two days standard culture [30]. Most previous studies on urinary microbiota have used metagenomic testing, which is expensive and time consuming and therefore usually not possible in an everyday clinical setting. Our use of two- and five-days culture without use of metagenomic testing makes the results applicable in everyday clinical practice. We also consider the use of extended microbiological protocol with prolonged aerobic and anaerobic incubation a strength of this study. This was chosen to increase detection of fastidious, slow-growing and anaerobic bacteria that are frequently missed by standard urine culture. Recent evidence indicates that conventional culture conditions underestimate bacterial presence in urine, and that extended culture methods can recover viable organisms with potential relevance for chronic or unclear urinary tract symptoms [14].

We also acknowledge as a weakness that the study was most likely underpowered to demonstrate statistically significant results for some of the outcomes. The study population was too small to perform any meaningful subgroup analysis of pre- and postmenopausal women, and we had no reliable information regarding use of hormones. Some of the women received antibiotic treatment after they were examined, but any potential benefit of this treatment on their symptoms was not assessed. We did not include information regarding development of systemic comorbidities, medication use and any subsequent pelvic or urological interventions, that may all influence both urinary microbiota and lower urinary tract symptoms independently of the original TVT surgery. All these factors increase with increasing age, and may add to the strong effect of age in this study population. Women were asked if they had persistent pain that occurred in relation to the TVT procedure, but it was not clinically verified by a health professional as sling-related or whether it had other causes, and thus subject to recall bias. A limitation of our approach is the use of a significance threshold derived from symptomatic UTI guidelines in a population with absent or non-specific symptoms. While this may increase the risk of classifying asymptomatic bacteriuria as significant growth, the threshold was chosen to explore whether low-grade bacteriuria detected by extended culture is associated with chronic symptoms rather than to imply infection. Another limitation is the classification of some of the bacterial species not reported in international literature with respect to uropathogenicity as doubtful pathogens and commensal genital flora. Established guidelines for interpretation originate from a time when species identification was performed only for the most common bacterial pathogens. Since the introduction of Maldi TOF MS technology and sequencing techniques, several more species can be identified in the diagnostic routine of the laboratory, although their role as commensal, doubtful pathogen or pathogen has not been fully resolved yet [30]. Lastly, the cross-sectional study design does not allow to determine any causality, only associations, between symptoms and the presence, amount and types of urinary bacteria.

## Conclusions

The sensitivity and positive predictive value of a positive urine dipstick test were 44% and 58% respectively, indicating that urine culture is necessary to diagnose significant bacteriuria or urinary tract infection more than 10 years after TVT, especially if women are dissatisfied, have incontinence symptoms or persistent pain. A larger variety of bacterial species after five days extended culture was associated with UUI, pain and dissatisfaction, and extended urinary culture may therefore be of value in women with UUI and pain after TVT.

## Supplementary Information

Supplementary Material 1: Table S1. Culture media, volumes, incubation time and atmosphere used in the present study. Table S2: Urinary tract pathogens (commonly found pathogens in bold).

## Data Availability

The datasets generated during and/or analyzed during the current study are available from the corresponding author on reasonable request.

## Abbreviations

TVT: Tension Free Vaginal Tape
SUI: Stress Urinary Incontinence
UUI: Urgency Urinary Incontinence
CFU/ml: Colony Forming Units per milliliter
Maldi: TOF MS–Matrix–Assisted Laser Desorption/Ionization Time–of–Flight Mass Spectrometry
BMI: Body Mass Index
SPSS: Statistical Package for the Social Sciences
