# Supplementary material for: Culture-detected urinary bacteria 10–20 years after tension-free vaginal tape surgery and associations with incontinence, pelvic pain, and dissatisfaction: a cross-sectional study
Source: BMC Urol. 2026 May 18;26:167. doi: 10.1186/s12894-026-02181-7 (PMC13371224; doi:10.1186/s12894-026-02181-7)
Supplement: Supplementary file 1 — Supplementary Material 1: Table S1. Culture media, volumes, incubation time and atmosphere used in the present study. Table S2: Urinary tract pathogens (commonly found pathogens in bold). [file 12894_2026_2181_MOESM1_ESM.docx]

**Table S1.** Culture media, volumes, incubation time and atmosphere used in the present study

| **Media** | **Inoculated**  **volume** | **Incubation time** | **Atmosphere** |
| --- | --- | --- | --- |
| Chromogenic agar  (Brilliance TM UTI Clarity TM, Oxoid) | 1 µL | 2 days | 35-37°C, normal atmosphere |
| Blood agar  (Columbia agar with 5% sheep blood, BD) | 10 µL | 2 days and 5 days | 35-37°C, 5% CO2 |
| Chocalate agar  (In-house) | 100 µL | 5 days | 35-37°C, 5% CO2 |
| Fastidious anaerobic agar  (Neogen) | 100 µL | 5 days | 35-37°C, anaerobic |

**Table S2:** Urinary tract pathogens (commonly found pathogens in bold)

| **Pathogens (primary)** | ***Escherichia coli***  ***Staphylococcus saprophyticus***  *Salmonella spp* |
| --- | --- |
| **Pathogens (secondary)** | ***Enterococcus spp***  ***Enterobacter spp***  ***Klebsiella spp***  ***Proteus mirabilis***  ***Pseudomonas aeruginosa***  *Citrobacter spp*  *Morganella morganii*  *Proteus vulgaris*  *Serratia spp*  *Staphylococcus aureus*  *Corynebacterium urealyticum*  *Haemophilus spp*  *Streptococcus pneumoniae* |
| **Doubtful pathogens** | *Streptococcus agalactiae*  *Yeast*  Coagulase negative Staphylococci  Othe*r Pseudomonas spp*  *Acinetobacter spp*  *Stenotrophomonas maltophilia*  *Aerococcus urinae* |
| **Genital flora/ non-pathogenic** | Other α-haemolytic *Streptococcus spp*  *Gardnerella spp*  *Lactobacillus spp*  *Bifidobacterium spp*  *Other Corynebacterium spp* |

*****Adapetd to English from National guidelines microbiological diagnosis of urinary tract infections (In Norwegian) <https://www.fhi.no/globalassets/dokumenterfiler/rapporter/strategirapporter/strategirapport-nr-21-2007-bakteriologisk-diagnostikk-ved-urinveisinfeksjon.pdf>

The list is not exhaustive, and findings in clinical urinary tract specimens will normally be related to both sampling method and clinical information (symptoms, underlying or predisposing conditions)
